# Second Annual Meeting of the Southern Section of the American Laryngological, Rhinological and Otological Society, Atlanta, Ga., March 28th, 1898

**Published:** 1898-03

**Authors:** 


### Society Reports.

### SECOND ANNUAL MEETING OF THE SOUTHERN SECTION OF THE AMERICAN LARYNGOLOGICAL, RHINOLOGICAL AND OTOLOGICAL SOCIETY, ATLANTA, GA., MARCH 28TH, 1898.

Session called to order at 9:15 A. M., in the Kimball House ballroom.

PROGRAM OF PAPERS.

1. Prayer.
2. Address of Welcome by the Mayor.
3. Response.
4. Address of the Chairman.—Dr. A. W. Calhoun, Atlanta, Ga.

(Twenty minutes for Registration.)

5. Thrombosis of the Lateral Sinus, Dependent upon Suppurative Otitis Media, with report of cases.—Dr. E. B. Dench, N. Y.
6. Labyrinthine Vertigo.—Dr. John Hey Williams, Asheville, N. C.
7. Chromatic Audition.—Dr. J. L. Minor, Memphis, Tenn.
8. Report of a Case of Hemorrhage after the Removal of Adenoid Vegetations.—Dr. Ross P. Cox, Rome, Ga.
9. Hypertrophy of the Lingual Tonsil, its Symptoms and its Treatment.—Dr. D. A. Kuyk, Richmond, Va.
10. Othæmatoma and Pericondritis of the Auricle.—Dr. John O. McReynolds, Dallas, Tex.
11. Tracheotomy for Foreign Bodies in the Air Passages, Report of Twenty-seven Successful Cases.—Dr. Willis F. Westmoreland, Atlanta, Ga.
12. A Form of Primary Nasal Diphtheria.—Dr. E. C. Ellett, Memphis, Tenn.
13. Empyema of the Accessory Nasal Cavities.—Dr. Ruffin A. Wright, Mobile, Ala.
14. Nasal Fibromata with Report of Cases.—Dr. L. M. Crichton, Atlanta, Ga.
15. The Influence on Development of the Nervous System of the Child by Adenoid Vegetations in the Naso-pharynx.—Dr. E. P. Sale, Memphis, Tenn.
16. The Importance of Examing the Nose in Troublesome Coughs.—Dr. Alfred C. Palmer, Richmond, Va.
17. Intubation of the Larynx for Membranous Stenosis.—Dr. Bernard Wolff, Atlanta, Ga.

18. Mental Disturbance in Turbinate Hypertrophies or Nasal Stenosis.—Dr. John F. Woodward, Norfolk, Va.

19. A Discovery in the Physiology of the Ear.—Dr. W. F. Cole, Waco, Tex.

20. Middle Ear Catarrh, Some Original Deductions.—Dr. Maury M. Stapler, Macon, Ga.

AFTERNOON SESSION—2:30 TO 5:30 P. M.

21. Presentation of a Cases with the Styloid Process in the Tonsil.—Dr. Alex. W. Stirling, Atlanta, Ga.

22. Ethmoiditis and Report of Cases.—Dr. B. F. Travis, Chattanooga, Tenn.

23. Mastoid Inflammation, with Report of Two Interesting Cases.—Dr. T. E. Mitchell, Columbus, Ga.

24. Mouth Breathing in Children, Particularly as a Result of Adenoids.—Dr. Arthur G. Hobbs, Atlanta, Ga.

25. The Naso-pharynx in Laryngeal Diseases.—Dr. N. C. Steel, Chattanooga, Tenn.

26. Cholesteatoma of the Mastoid Antrum.—Dr. S. L. Ledbetter, Birmingham, Ala.

27. Clinical Miscellanies from Private Practice.—Dr. Frank M. Mullins, Ft. Worth, Tex.

28. Empyema of the Accessory Sinuses of the Nose.—Dr. Frank M. Hanger, Staunton, Va.

29. A Good Hemostatic after Nasal Operations.—Dr. L. B. Grandy, Atlanta, Ga.

30. Hypertrophic Rhinitis and its Treatment.--Dr. J. F. Hill, Memphis, Tenn.

31. The Specialist and the Public Health.—Dr. A. T. Mitchell, Vicksburg, Miss.

32. The Cure of Nasal Hypertrophy by Gentle Dilatation, and Report of Cases.—Dr. George Horine, Americus, Ga.

33. The Serum Treatment of Ozena.—Dr. W. E. Campbell, Atlanta, Ga.

34. Some Remarks upon Syphilitic Manifestations in the Larynx.—Dr. P. T. Vaughan, Hot Springs, Ark.

35. Screw Worms in the External Auditory Canal.—Dr. R. F. Miller, Sherman, Tex.

EVENING SESSION—7:30 P. M. TO 9 P. M.

36. The Abortive Treatment of Acute Tonsilitis and Laryngitis.—Dr. E. P. Davis, Houston, Tex.

37. Peritonsillar Abscess.—Dr. Dunbar Roy, Atlanta, Ga.

38. The Influence of diseases of the Accessory Cavities on the General Health.—Dr. W. Scheppegrell, New Orleans, La.

39. Operations in the Middle Ear, Fifty Consecutive Cases and Results.—Dr. J. A. Stucky, Louisville, Ky.

40. Adenoid Vegetations in the Vault of the Pharynx with Report of Cases.—Dr. Frank D. Boyd, Ft. Worth, Tex.

41. The Treatment of Chronic Suppuration of the Attic.—Dr. W. S. Sims, Meridian, Miss.

42. A Case of Fibroma of the Naso-pharynx with Remarks.—Dr. L. G. Woodson, Birmingham, Ala.

43. Politzeration in Acute Otitis Media.—Dr. R. H. Chilton, Dallas, Tex.

44. Reflex Aural Disorders.—Dr. A. G. Sinclair, Memphis, Tenn.

45. Vibratory Massage in the Treatment of Atrophic Rhinitis.—Dr. Richmond McKinney, Memphis, Tenn.

46. The Advantages of Operations in Suppurative Troubles of the Middle Ear.—Dr. J. H. Shorter, Macon, Ga.

47. Diseases of the Accessory Sinuses.—Dr. Alex. W. Stirling, Atlanta, Ga.

48. The Control of Nasal Hemorrhage.—Dr. J. M. Crawford, Atlanta, Ga.

9:15 P. M.

Reception tendered to the Fellows of the Society and to the Visiting Physicians by Dr. A. W. Calhoun at his residence, 672 Peachtree street.

The Reading of Papers will be limited to twenty minutes each.

Discussion of a Paper will be limited to five minutes each.

Both the Kimball House and Aragon Hotel have given reduced rates.

The Medical Profession is cordially invited.

